# Real-time assessment of the behaviour of the bladder neck and proximal urethra during urine leaking in the cough stress test (CST) in supine and standing positions using transperineal ultrasound

**DOI:** 10.1007/s00192-020-04273-w

**Published:** 2020-04-14

**Authors:** Lieming Wen, Baihua Zhao, Wenjie Chen, Zhenzhen Qing, Minghui Liu

**Affiliations:** 1 Department of Ultrasound Diagnosis, The Second Xiangya Hospital of Central South University, 139 Renmin Road (M), Changsha, 410011 Hunan China; 2 The First Affiliated Hospital of Hunan University of Chinese Medicine, Changsha, China

**Keywords:** Stress urinary incontinence, Cough stress test, Pelvic floor ultrasound, Urethral mobility

## Abstract

**Introduction and hypothesis:**

The objective was to describe the behaviour of the bladder neck and proximal urethra during urine leaking in the cough stress test (CST) in supine and standing positions using transperineal ultrasound (TPUS).

**Method:**

We carried out prospective data collection and a retrospective data analysis of 102 women with stress urinary incontinence (SUI) who had a positive CST with TPUS in the supine and/or standing position. On TPUS, the behaviour of the bladder neck and proximal urethra was described by the urethral length, urethral funnelling, bladder neck descent (BND), retrovesical angle (RVA) and urethral rotation angle (URA). Differences between the ultrasound findings in the two positions were evaluated.

**Results:**

In the 102 women, the mean age was 48 years and mean BMI was 23.8 kg/m^2^. On TPUS, urine leakage was detected in the supine or standing position in 102 women and in both positions in 81. Between the two positions, significant differences were found in the URA and RVA. In the standing position, the median RVA of 166° was significantly larger than that of 133° in the supine position (*p* < 0.001), and the median URA of 35° was significantly smaller than that of 64° in the supine position (*p* < 0.001).

**Conclusions:**

TPUS in both positions can be used to detect the real-time behaviour of the bladder neck and urethra in the CST. In the standing position, less rotation and more straightening of the bladder neck and proximal urethra occurred during urine leakage.

**Electronic supplementary material:**

The online version of this article (10.1007/s00192-020-04273-w) contains supplementary material, which is available to authorized users.

## Introduction

Female stress urinary incontinence (SUI) is defined by the International Continence Society (ICS) as leakage that results from increased abdominal pressure. It has a high prevalence of 20% [1]. The cough stress test (CST) applies a simple stress or Valsalva manoeuvre to induce leakage. Many studies have shown that the cough stress test (CST) is a conservative and non-invasive diagnostic technique that can be easily employed in a clinic setting with high sensitivity and specificity [2, 3]. Several clinical practice guidelines have recommended the performance of a CST for the diagnosis of SUI [4, 5]. Urethral hypermobility is considered to be a main underlying pathological factor for SUI [6, 7]. Evaluating the behaviour of the bladder neck and urethra in the CST is vital for improving our understanding of SUI. Anatomical changes in women with SUI, such as bladder neck mobility, funnelling and urethral mobility, have been evaluated [8, 9]. However, there are limited data regarding the real-time anatomical changes that occur during urine leaking in the CST.

Ultrasound with colour Doppler has been used as a non-invasive method to observe urine leakage for 20 years [10, 11]. The quality of colour Doppler imaging may be affected by many factors such as the velocity scale and fluid speed. It has not been commonly used to observe real-time leakage. However, with the improvement of ultrasonic image resolution, the urethral structure can now be observed in detail [12]. This allows us to distinguish the anechoic urine and the hypoechoic urethral mucosa. It may be possible to observe leakage in the CST and assess the configuration and mobility of the bladder neck and urethra in real time with US imaging.

In this study, we aimed to evaluate the use of transperineal ultrasound (TPUS) for detecting real-time urine leakage in the CST in women with SUI and to compare differences in the anatomical changes of the bladder neck and proximal urethra in the CST between the supine and standing positions.

## Materials and methods

This was a retrospective study of 102 women with clinical SUI who had a positive CST using TPUS in the supine and/or standing positions in the Second Xiangya Hospital between November 2018 and May 2019. The data collection for this study was approved by the Human Research Ethics Committee of the Second Xiangya Hospital (no. 2019–041).

All women had a standard interview and a CST. The CST was performed approximately half an hour after voiding, with the bladder containing < 50 ml urine. The patient coughed forcefully 1–4 times and the examiner directly visualized the urethral meatus for the presence of leakage. Leakage of fluid from the urethral meatus occurring simultaneously with the coughs was considered a positive test [3]. Clinical SUI was diagnosed if a patient presented with involuntary urine leakage from physical activity such as coughing, sneezing or laughing and had a positive CST. Based on the interview, the severity of urinary incontinence was classified using the Ingelman-Sundberg scale [13], including grade I: urinary incontinence when coughing or sneezing; grade II: urinary incontinence when running or picking up items from the floor; grade III: urinary incontinence when walking or climbing stairs.

None of the women had any history of: (1) previous pelvic or pelvic floor surgery or physiotherapeutic interventions; (2) pain; (3) haematuria; (4) recurrent infections; (5) voiding symptoms; (6) pelvic irradiation; (7) suspected fistulas; (8) pelvic organ prolapse beyond Stage 2 of the Pelvic Organ Prolapse Quantification System.

**Fig. 1:**
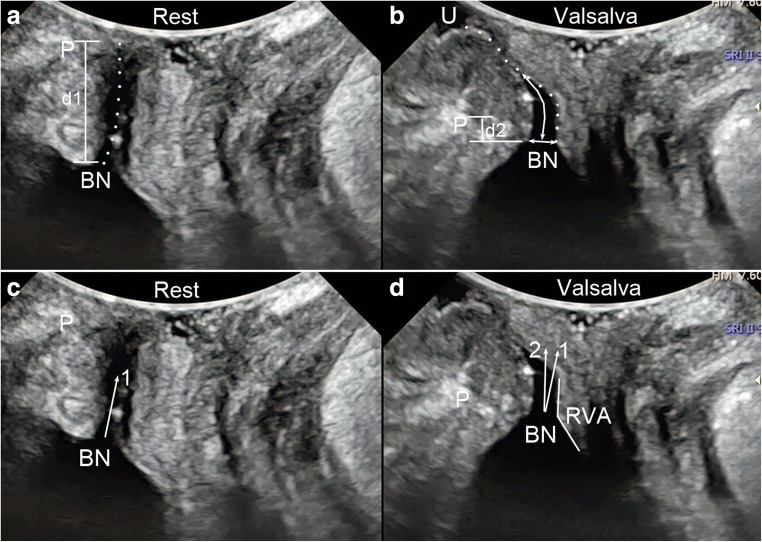
Transperineal ultrasound images in the mid-sagittal plane during real-time leaking. A and C were taken at rest, B and D during the Valsalva manoeuvre. **a** and **b** Urethral movement during the Valsalva manoeuvre; the dotted line in A shows the urethra at rest while in B it shows the urethra during the Valsalva manoeuvre. The length of the dotted line is equal to the urethral length. **b** Funnelling measurements (the long arrow shows the funnelling length and the short arrow shows the funnelling width) and urine leakage, seen as fluid (urine) between the external urethral orifice and the probe surface. **a** and **b** Measurement of the position of the bladder neck relative to the posterior inferior margin of the pubic symphysis (P); the difference between these measurements during the peak of the Valsalva manoeuvre and at rest (d1–d2) produces the value for bladder neck descent (BND). **c** and **d** Measurement of the urethral rotation angle (URA), which is the angle between the proximal urethra at rest (arrow 1) and during the Valsalva manoeuvre (arrow 2). **d** The retrovesical angle (RVA) during the Valsalva manoeuvre, which is measured between the proximal urethra and the trigone. P: symphysis pubis, BN: bladder neck, URA: urethral rotation angle, RVA: retrovesical angle, U: urine

**Fig. 2:**
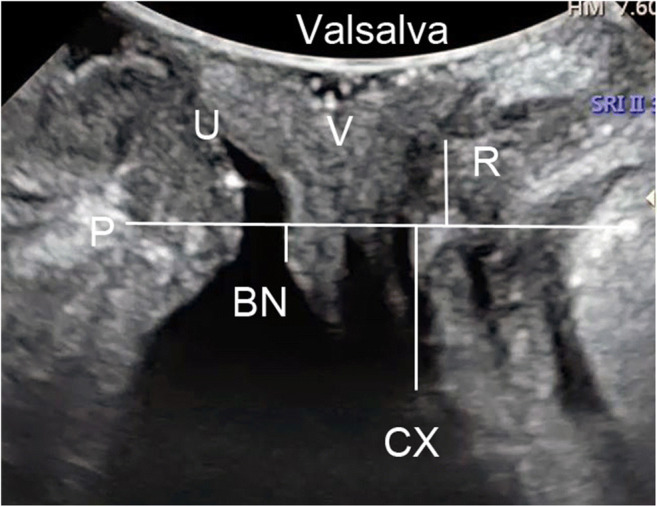
Transperineal ultrasound image in the mid-sagittal plane demonstrates the descent of the bladder, uterus and rectal ampulla during real-time leaking in the cough stress test. Measurements represent the maximally caudal organ position, which is the distance between the leading edge of the organs [bladder neck (BN), cervix (CX) and rectal ampulla (R)] and the horizon line at the level of the posterior inferior margin of the pubic symphysis (P). Measurements cranial to the reference line are positive; those caudal to the line are negative. P: pubic symphysis, BN: bladder neck, CX: cervix, U: urethra, V: vagina, R: rectal ampulla

TPUS was performed followed by CST with the bladder filled with < 50 ml urine using a Voluson E8 or E10 system (GE Healthcare, Milwaukee, WI, USA) or a Resona 7 or 8 system (Mindray Medical International, Shenzhen, China) equipped with a 4–8-MHz curved array transducer placed on the perineum in the sagittal direction [14]. The acquisition angle was set to the system maximum of 85°. Volumes were acquired during the Valsalva manoeuvre, which lasted until urine leakage was detected on the image. Leakage was defined as urine being detected in the urethral tract or between the external urethral orifice and the probe surface (Fig. 1b). Images of at least three Valsalva manoeuvres per patient were acquired in both the supine and standing positions. Post-processing analysis was later performed by two researchers (Zhao and Wen), who were blinded to the clinical findings. The bladder neck descent, urethral rotation angle, retrovesical angle, urethral length, urethral funnelling, and funnelling width and length during leakage were measured using the image that showed real-time urine leaking (Fig. 1). Urethral funnelling was defined if the urethral internal orifice was opened during the Valsalva manoeuver. Funnelling width was the anterioposterior diameter of the orifice. Funnelling length was the length of the opened urethra (Fig. 1). The maximally caudal organ positions (of the bladder neck, uterus and rectal ampulla) relative to the pubic symphysis were also measured (Fig. 2).

Statistical analyses were undertaken using SPSS v.17 (SPSS, Inc., Chicago, IL, USA). Normality of the continuous data was assessed using the Kolmogorov-Smirnov test. US findings in the supine and standing positions were compared using the paired t-test for normally distributed data and the Wilcoxon test for non-normally distributed data. A *p* value < 0.05 was considered statistically significant.

A test-retest series with a total of 20 patients was performed on two different raters looking at the same ultrasound image. The intraclass correlation coefficients (ICCs) of the inter-rater agreement for detecting the maximally caudal positions of the bladder neck, uterus and rectal ampulla were 0.89 (95% CI: 0.81–0.96), 0.82 (95% CI: 0.73–0.93) and 0.77 (95% CI: 0.63–0.92), respectively. The ICC was 0.86 for the bladder neck descent (95% CI: 0.78–0.96), 0.92 for the urethral rotation angle (95% CI: 0.85–0.97), 0.91 for the retrovesical angle (95% CI: 0.83–0.97), 0.83 for the urethral length during the Valsalva manoeuvre (95% CI: 0.75–0.93) and 0.95 for the urethral funnelling measurements (95% CI: 0.83–0.98). The ICC for detecting leakage was 0.91 (95% CI: 0.83–0.98).

## Results

In the 102 women with clinical SUI, the mean age was 48.2 ± 8.7 years (range: 28.0–72.0), mean BMI was 23.8 ± 2.9 kg/m^2^ (range: 18.7–33.3), and 95% of the women (*n* = 97) were vaginally parous with a mean parity of 2 ± 1 (range: 1–6). The mean age at first childbirth was 23.2 ± 3.5 years (range: 18.0–34.0), and the mean interval between first childbirth and first visit to a doctor for urine leakage was 20.2 ± 10.0 years (range: 0.0–51.5).

On the clinical interviews, the mean history of leakage was 5.8 ± 5.5 years (range: 0.1–30.0). According to the Ingelman-Sundberg scale, 43 women (42.2%) had Grade I SUI, 42 (41.2%) had Grade II and 17 (16.7%) had Grade III. On TLUS, real-time leaking was detected in the supine or standing position in all 102 women. However, a poor US image was captured in the standing position for eight women (7.8%). Of the remaining 94 women, urine leakage was detected only in the standing position for 13 (13.8%). This left 81 women with leakage detected in both positions for the comparison study. This included 31 women (38.3%) with Grade I SUI, 36 (44.4%) with Grade II SUI and 14 (17.3%) with Grade III SUI. Details are shown in Table 1.

**Table 1:** Details of the study trial

Clinical findings	Total number	Poor image	No leakage in supine position	Valid data
SUI Grade I	43 (100%)	2 (4.7%)	10 (23.8%)	31(72.1%)
SUI Grade II	42 (100%)	3 (7.1%)	3(7.1%)	36 (85.8%)
SUI Grade III	17 (100%)	3 (17.6%)	0 (0.0%)	14 (82.4%)
Total	102 (100%)	8 (7.9%)	13(12.7%)	81 (79.4%)

According to the ultrasound images, during real-time leaking in the supine position, the maximally caudal position of the bladder neck, uterus and rectal ampulla relative to the pubic symphysis were 0.49 ± 7.91 mm, 16.93 ± 11.78 mm and − 5.91 ± 9.69 mm, respectively. In the standing position, the maximal caudal positions of the bladder neck, uterus and rectal ampulla were 1.33 ± 7.95 mm, 17.89 ± 10.48 mm and − 3.10 ± 10.87 mm, respectively. There were no significant differences in the maximally caudal positions of the bladder (*p* = 0.282) or uterus (*p* = 0.382) between the two positions. A significant difference was found in the maximal caudal position of the rectal ampulla (*p* = 0.034).

Data regarding the configuration and mobility of the bladder neck and proximal urethra of all 81 women in the CST in both positions are shown in Table 1. No significant differences in bladder neck descent, urethral funnelling and funnelling width and length or urethral length during leakage were found between the two positions. However, significant differences were found in the retrovesical angle and urethral rotation angle between the two positions (Table 2). In the standing position, the RVA was significantly increased (*p* < 0.001) and the urethral rotation angle was significantly decreased (*p* < 0.001) relative to those in the supine position.

**Table 2:** Configuration and mobility of the bladder neck and proximal urethra in the CST in the supine and standing positions (*N* = 81)

US findings	CST in the supine position	CST in the standing position	*p* value
BND^a^	27.01 ± 8.50	25.78 ± 9.08	0.088
Retro-vesical angle^b^	133 (119, 148)	166 (153, 177)	0.000
Rotation angle^b^	64 (44, 81)	35 (28, 41)	0.000
Funnelling (*n*, %)^c^	37 (45.68%)	39 (44.83%)	–
Funnelling width^a^	6.21 ± 2.33	6.10 ± 1.82	0.735
Funnelling length^a^	11.37 ± 4.54	11.38 ± 3.35	0.985
Urethral length^a^	39.35 ± 6.94	38.06 ± 5.63	0.136

^a^Values are expressed as (mean ± SD) mm; *p* values are yielded by paired t-test. ^b^Values are expressed as median (IQR: Q1, Q3); *p* values are yielded by Wilcoxon ranks test. ^c^Kappa value is 0.802 (*p* < 0.001) yielded by chi-square test

## Discussion

Many studies have shown that the CST is a non-invasive and easy-to-perform clinical test for the diagnosis of SUI [3]. A recent report showed that the CST has high sensitivity and specificity at different bladder fill volumes [2]. Studies using clinical examinations and US imaging in the supine position have indicated that aspects of the configuration and mobility of the bladder neck and urethra, such as bladder neck hypermobility, UF and urethral length, are significantly correlated with SUI [8, 9]. Actually, normal continent women may have significant mobility of the urethra. Studies comparing cases and asymptomatic controls have shown that there is a major overlap in urethral support between women with and without SUI [15]. It is not possible for examiners to know whether or not someone has SUI based simply on ultrasound images that do not include visualization of leakage [16, 17]. Therefore, assessment of the real-time behaviour of the bladder neck and proximal urethra during leakage in the CST is very important for understanding the underlying anatomic mechanisms of incontinence.

In this study, no significant differences in the maximally caudal organ positions were found in the anterior and middle pelvic compartments during leakage resulting from the Valsalva manoeuvre. This was consistent with the findings of a previous report that assessed pelvic floor prolapse using TPUS in the supine and standing positions [18]. Real-time urine leakage in the CST was detected using TPUS in both the supine and standing positions. The bladder neck descent, urethral length and urethral funnelling resulting from stress in the CST were the same in the two positions.

However, in the standing position, the urethral rotation angle was significantly decreased and the retrovesical angle was significantly increased compared with those in the supine position. This indicated that the movement of the bladder neck and proximal urethra was different between the two positions. More dorsal movement occurred in the supine position, while more straightening occurred in the standing position during urine leakage in women with SUI. It is plausible to speculate that the approach and direction of the intra-abdominal pressure transmission were different between CST in the supine position and real-time leakage in the standing position in everyday life. The symptoms and signs presented in the standing position may be more credible in SUI assessment. An obliterated retrovesical angle may play an important role in the mechanisms of incontinence. This point was similar to the findings of radiological studies performed 60 years ago [19, 20] and was different from previous US studies [14, 16, 17, 21]. This may affect the investigation of urethral mobility and the treatment of SUI. However, proving this requires a series of follow-up studies. Previous work showed that a certain anatomical configuration of the bladder neck and trigone was associated with the urodynamic diagnosis of USI, but it was not sufficient to allow a ‘prediction’ of USI on imaging [17]. These findings on urethral support and funnelling need to be put into the context of what we know about stress incontinence. The differences between the real-time anatomical changes of the bladder neck and urethra in the CST between women with and without SUI should be addressed in future work.

The more complex four-dimensional TPUS was used in our study to obtain more accurate and reliable measurements. However, all the parameters included in our analysis were measured in the pelvic floor mid-sagittal plane (Figs. 1 and 2). This means that one could evaluate the configuration and mobility of the bladder neck and urethra in the CST using two-dimensional TPUS, which is inexpensive and easy to perform using a common two-dimensional abdominal US system [8, 9, 14]. It is clear that a CST with TPUS allows clinicians to obtain more important information during an assessment for SUI. This can be easily used in clinical practice because it is non-invasive and easily accessible.

There is no doubt that there were many limitations to this study that need to be acknowledged. First, the SUI diagnoses and CST and TPUS findings were not connected to the urodynamic findings such as MUCP and urodynamic stress incontinence. Second, in this study, US and CST were performed on bladder volume < 50 ml to obtain a high-quality ultrasound images and accurate measurements. Ultrasound and CST performed on different bladder volumes might have different results. Third, urethral funnelling has not been defined in detail according to the relationship between urethral funnelling and SUI [21]. Whether there was funnelling or not was a subjective assessment. Fourth, this was a single-centre study and only included Chinese women. The reproducibility and reliability of the standing TPUS need to be further studied. Similar studies in other racial groups should be performed.

## Conclusions

TPUS in both the supine and standing positions can be used to detect the real-time behaviour of the bladder neck and proximal urethra during urine leakage in the CST. In the standing position, less dorsal movement and more straightening occurred in the bladder neck and proximal urethra during urine leakage in women with SUI. The significance of this phenomenon for SUI investigation needs to be further studied.

## Electronic supplementary material

ESM 1 (MP4 1513 kb)

ESM 2 (MP4 1301 kb)

ESM 3 (MP4 1776 kb)

## References

[1] Rubilotta E, Balzarro M, D'Amico A (2019). Pure stress urinary incontinence: analysis of prevalence, estimation of costs, and financial impact. BMC Urol.

[2] Henderson JW, Kane SM, Mangel JM (2018). A randomized comparative study evaluating various cough stress tests and 24-hour pad test with urodynamics. J Urol.

[3] Guralnick ML, Fritel X, Tarcan T, Espuna-Pons M, Rosier PFWM (2018). ICS educational module: cough stress test in the evaluation of female urinary incontinence: introducing the ICS-uniform cough stress test. Neurourol Urodyn.

[4] No O (2014). 603: evaluation of uncomplicated stress urinary incontinence in women before surgical treatment. Obstet Gynecol.

[5] Dmochowski RR, Blaivas JM, Gormley EA (2010). Female stress urinary incontinence update panel of the American Urological Association Education and Research, Inc, Whetter LE. Update of AUA guideline on the surgical management of female stress urinary incontinence. J Urol.

[6] Delancey JO (2010). Why do women have stress urinary incontinence?. Neurourol Urodyn.

[7] Lukacz ES, Santiago-Lastra Y, Albo ME, Brubaker L (2017). Urinary incontinence in women: a review. JAMA..

[8] Naranjo-Ortiz C, Shek KL, Martin AJ, Dietz HP (2016). What is normal bladder neck anatomy?. Int Urogynecol J.

[9] Wlaźlak E, Kluz T, Surkont G, Kociszewski J (2018). Urethral funneling visualized during pelvic floor sonography-analysis of occurrence among urogynecological patients. Ginekol Pol.

[10] Dietz HP, Clarke B (2001). Translabial color doppler urodynamics. Int Urogynecol J Pelvic Floor Dysfunct.

[11] Dietz HP, McKnoulty L, Clarke B (1999). Translabial color Doppler for imaging in urogynecology: a preliminary report. Ultrasound Obstet Gynecol.

[12] Zacharakis D, Grigoriadis T, Pitsouni E, Domali E, Protopapas A, Athanasiou S (2017). Ultrasonographic evaluation of the urethral rhabdosphincter morphology in female patients with urodynamic stress incontinence. Female Pelvic Med Reconstr Surg.

[13] Ingelman-Sundberg A, Ulmsten U (1983). Surgical treatment of female urinary stress incontinence. Contrib Gynecol Obster.

[14] Dietz HP (2004). Ultrasound imaging of the pelvic floor: part 1: 2D aspects. Ultrasound Obstet Gynecol.

[15] DeLancey JO, Trowbridge ER, Miller JM (2008). Stress urinary incontinence: relative importance of urethral support and urethral closure pressure. J Urol.

[16] Lewicky-Gaupp C, Blaivas J, Clark A (2009). "the cough game": are there characteristic urethrovesical movement patterns associated with stress incontinence?. Int Urogynecol J Pelvic Floor Dysfunct.

[17] Wlaźlak E, Surkont G, Shek KL, Dietz HP (2015). Can we predict urinary stress incontinence by using demographic, clinical, imaging and urodynamic data?. Eur J Obstet Gynecol Reprod Biol.

[18] Braverman M, Kamisan Atan I, Turel F, Friedman T, Dietz HP (2019). Does patient posture affect the ultrasound evaluation of pelvic organ prolapse?. J Ultrasound Med.

[19] Jeffcoate TN, Roberts H (1952). Observations on stress incontinence of urine. Am J Obstet Gynecol.

[20] Aldridge A, Jeffcoate TN, Roberts H (1952). Stress incontinence of urine. J Obstet Gynaecol Br Emp.

[21] Dietz HP, Nazemian K, Shek KL, Martin A (2013). Can urodynamic stress incontinence be diagnosed by ultrasound?. Int Urogynecol J.

